# Simultaneous Measurement of Gastric Emptying of a Soup Test Meal Using MRI and Gamma Scintigraphy

**DOI:** 10.3390/diagnostics10030170

**Published:** 2020-03-22

**Authors:** Asseel Khalaf, Caroline L. Hoad, Elaine Blackshaw, Jaber Alyami, Robin C. Spiller, Penny A. Gowland, Vidhiya Vinayaka-Moorthy, Alan C. Perkins, Gordon W. Moran, Luca Marciani

**Affiliations:** 1 Radiologic Sciences, Allied Health Sciences, Kuwait University, 90805 Sulaibekhat, Kuwait City, Kuwait; asseel.khalaf@hsc.edu.kw; 2 Nottingham Digestive Diseases Centre and National Institute for Health Research (NIHR) Nottingham Biomedical Research Centre, Nottingham University Hospitals NHS Trust and University of Nottingham, Nottingham NG7 2UH, UK; caroline.l.hoad@nottingham.ac.uk (C.L.H.); robin.spiller@nottingham.ac.uk (R.C.S.); penny.gowland@nottingham.ac.uk (P.A.G.); gordon.moran@nottingham.ac.uk (G.W.M.); 3 Sir Peter Mansfield Imaging Centre, School of Physics and Astronomy, University of Nottingham, Nottingham NG7 2QX, UK; 4 Medical Physics and Clinical Engineering, Nottingham University Hospitals NHS Trust, Nottingham NG7 2UH, UK; Elaine.Blackshaw@nottingham.ac.uk (E.B.); Vidhiya.Vinayaka-Moorthy@nuh.nhs.uk (V.V.-M.); 5 Diagnostic Radiology, Faculty of Applied Medical Science, King Abdulaziz University, 21589 Jeddah, Saudi Arabia; jhalyami@kau.edu.sa; 6 Radiological Sciences, School of Medicine, University of Nottingham, Nottingham NG7 2UH, UK

**Keywords:** magnetic resonance imaging, stomach, validation, volumes, half emptying, activity

## Abstract

Measurement of gastric emptying is of clinical value for a range of conditions. Gamma scintigraphy (GS) has an established role, but the use of magnetic resonance imaging (MRI) has recently increased. Previous comparison studies between MRI and GS showed good correlation, but were performed on separate study days. In this study, the modalities were alternated rapidly allowing direct comparison with no intra-individual variability confounds. Twelve healthy participants consumed 400 g of Technetium-99m (^99m^Tc)-labelled soup test meal (204 kcal) and were imaged at intervals for 150 min, alternating between MRI and GS. The time to empty half of the stomach contents (T_1/2_) and retention rate (RR) were calculated and data correlated. The average T_1/2_ was similar for MRI (44 ± 6 min) and GS (35 ± 4 min) with a moderate but significant difference between the two modalities (*p* < 0.004). The individual T_1/2_ values were measured, and MRI and GS showed a good positive correlation (*r* = 0.95, *p* < 0.0001), as well as all the RRs at each time point up to 120 min. Gastric emptying was measured for the first time by MRI and GS on the same day. This may help with translating the use of this simple meal, known to elicit reliable, physiological, and pathological gastrointestinal motor, peptide, and appetite responses.

## 1. Introduction

The importance of measuring gastric emptying in clinical and research arenas has long been recognised for a range of conditions including gastroparesis [1], dyspepsia [2] and dumping syndrome [3]. Various techniques have been used over the years to measure gastric emptying. Methods such as dilution-intubation techniques [4], paracetamol absorption tests [5], stable isotope breath tests [6] and ultrasound [7] have limitations. Gamma scintigraphy (GS) [8] has gained an established role and is very often considered the ‘gold standard’ for the quantitative assessment of gastric emptying [9]. The assessment of gastric emptying using magnetic resonance imaging (MRI) [10] has, however, recently increased [11,12] due to its lack of use of ionizing radiation, multi-planar ability, spatial resolution and richness of contrast mechanisms [13].

Gastric MRI and GS imaging inherently have different physical reporter parameters. MRI is based on the radio frequency signal arising from the spatial distribution of water hydrogen protons in a test meal; GS is based on the detection of gamma photons emitted by a radiolabelled test meal. These physical differences indicated a need to compare the gastric emptying parameters obtained with the two techniques. Historical comparison studies between MRI and GS have shown good correlations between the two modalities [10,14], although these studies were performed on separate days and in a limited number of subjects. In more recent literature, there has been renewed interest in comparing the two techniques for gastric emptying. Two studies have shown a good correlation between the two modalities, once the additional contribution of gastric secretion volume was considered [15,16]. Another recent study of gastrointestinal complications after lung transplantation found no significant differences between gastric emptying parameters measured with MRI and GS [17].

These studies were, however, necessarily performed on two separate study days. This was likely to add potential intra-individual confounds due to physiological and environmental variations and did not allow for a direct comparison of gastric emptying curves acquired on the same study day.

In our research facilities, we have an MRI scanner unit located next to a gamma camera unit. In this study, we exploited the physical proximity of the two modalities. We aimed to evaluate the correlation of gastric emptying assessments between MRI and GS performed simultaneously on the same day in the same healthy participants. Alternating image acquisitions were performed using the two modalities throughout the emptying of a test meal. 

## 2. Materials and Methods

### 2.1. Subjects

Twelve healthy participants (age 21.6 ± 0.4 years, BMI 23.9 ± 0.9 kg/m^2^) were recruited from the local campus population by poster advertisement from January 2018 to March 2018. Participants with a history of bowel disease, smoking, a history of bowel resections or any gastric surgery, a history of pancreatic insufficiency, thyroid disease, diabetes, proton-pump inhibitor usage or any medication that affects gastric emptying were excluded. Standard MRI exclusion criteria were applied. This study was approved on 2^nd^ November 2017 by the UK Administration of Radioactive Substances Advisory Committee (ARSAC approval RPC 253/3849/37292), the Research Ethics Committee (REC approval 17/EM/0151) and the Health Research Authority (Protocol Number 17016). Informed written consent was obtained from all individual participants included in the study.

### 2.2. Test Meal

The test meal consisted of cream of chicken soup (400 g) (or mushroom for vegetarians) (Heinz, Wigan, UK) as used previously [18,19,20]. The nutrient content of this meal (100 g) was as follows: energy (kcal) 51, protein 1.5 g (1.5%), carbohydrate 4.7 g (4.5%) and fat 2.9 g (2.9%). This meal was previously shown to induce reliably gastrointestinal motor and peptide responses [19,20]. 

The soup was radiolabelled with 5 MBq of technetium-99 labelled non-absorbable marker (^99m^Tc-DTPA), which resulted in an effective radiation dose of 0.125 mSv. This dose and radiolabelling methods have been used previously by our group in previous healthy volunteer studies [16]. Radiopharmaceuticals (^99m^Tc-DTPA) were supplied by the local radiopharmacy unit and produced under good manufacturing practice (GMP) conditions. The addition of the radiolabel to the meal was carried out by authorised ARSAC operators. The radiolabelled test meal was administered to the participants orally on the morning of the study following an overnight fast, and details of ingestion of the meal were observed and recorded.

### 2.3. Study Design

This was a single-centre, open-label design study. Participants were asked to fill in a questionnaire to ensure adherence to the study day restrictions. The participants were asked to fast from 20:00 h the previous evening and to avoid alcohol, caffeine, strenuous exercise and any medication that could affect gut function for 18 h before the experiment. 

They were asked to change their clothing into disposable scrubs and to remove all metal objects (e.g., watches, jewellery). A baseline measurement was taken by MRI, which also ensured that the participants had a fasting stomach at baseline. The participants were then transported rapidly by wheelchair to the adjacent gamma scintigraphy unit. Small radioactive markers were affixed to the participants at the right costal margin, both anteriorly and posteriorly to enable accurate alignment, interpretation and quantification of the GS images. The participants were then asked to consume the radiolabelled soup test meal within 20 min and the first image was taken with the gamma scintigraphy immediately after that.

The participants were transported quickly by wheelchair back to the adjacent MRI unit where an MRI data point was acquired. Subsequently, the participants were then transported back to the GS unit and this was repeated approximately every 20 min for a total duration of 150 min, which previous studies suggested was the time by which most participants would have emptied the meal from the stomach. As a result, 6 data points per modality, per subject were collected.

### 2.4. MRI Protocol and Data Analysis

MRI imaging was performed in the supine position using a 1.5T GE Signa HDx MRI scanner (GE Healthcare, Milwaukee, WI, USA). At each time point, scans were acquired using a balanced steady-state gradient echo sequence (FIESTA) with a slice thickness of 5 mm, echo time (TE) = 1.2 ms, repetition time (TR) = 3.5 ms, flip angle 80° and within one breath hold of ~16 s. This imaging sequence yields good contrast between the stomach contents and other abdominal organs.

Gastric volumes from the MRI datasets were quantified using in-house software written in Interactive Data Language Visualization Solution (IDL) 8.5 software (Research Systems Inc. Boulder, CO, USA) [15]. This method uses a semi-automatic outlining of the stomach contents and air on each image slice using an intensity-based method to define both high signal intensity gastric content volume (GCV) and low signal intensity air. The total gastric volume (TGV) was calculated from the sum of the air and content regions. The segmented area on each slice was multiplied by the slice thickness and summed over all contoured slices to measure the different stomach volumes (TGV and GCV). Gastric content volumes were fitted to a five-parameter equation [16] to model the emptying process and to calculate gastric half emptying time (T_1/2_). As with [17], the retention rates RR (%) of gastric content volumes were also calculated as the percentage of GCV in the stomach at each time point relative to the GCV measured at time 0.

### 2.5. GS Protocol and Data Analysis

For the GS part of the study, a Mediso Gamma Camera (Nucline X-Ring-R, Budapest, Hungary) was used as described previously [16]. Two small ^99m^Tc radiolabelled markers (each containing 0.25 MBq) were affixed to the right anterior and posterior lower costal margins of the participants for the duration of the gastric emptying study. At each time point, the participants stood in front of the gamma camera and anterior and posterior images, each of a 30 s duration, were recorded.

**Figure 1:**
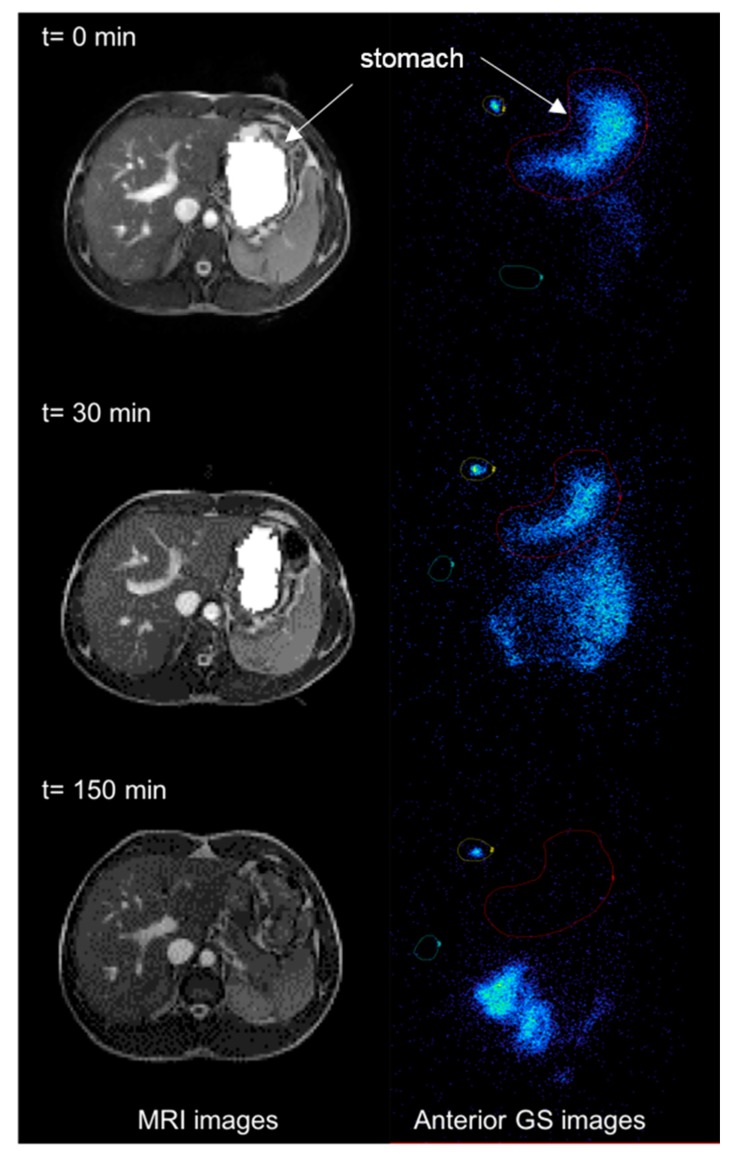
Example of magnetic resonance imaging (MRI) and Gamma scintigraphy (GS) images of the stomach at different time points after a participant ate the soup test meal (t = 0 immediately after feeding, t = 30 min after feeding and t = 150 min at the end of the study period). The axial, moderately T2 weighted images are shown on the left hand of the panel and the coronal planar gamma scintigraphy images are shown on the right hand of the panel, with labels and arrows to indicate anatomical landmarks. Regions of interests (ROIs) were defined around the labelled meal in the stomach (red), around an area of background activity (green) and around the radioactive marker (yellow).

Gastric volumes were analysed using methods described previously [15]. Briefly, to measure gastric emptying by GS, regions of interest (ROIs) were defined around the image of the meal in the stomach and around an area of background activity in the anterior and posterior images [16]. All counts were corrected for background radiation and radioactive decay (Figure 1). The values for each participant at each time point were then expressed as the percentage of activity relative to the counts obtained from the stomach ROI at time 0. The same process was repeated for all subsequent scintigraphic images. T_1/2_ was then calculated using the same equation fit as for the MRI data.

### 2.6. Statistical Analysis

Data analysis was performed using GraphPad Prism version 7.01 (GraphPad Software Inc., La Jolla, CA, USA). Results are presented as mean ± SEM. All data were tested for normality using the D’Agostino and Pearson normality test. A paired *t*-test was used to assess the significance of differences in T_1/2_. Pearson’s (or Spearman’s depending on normality) correlation coefficient was used to measure the strength of correlation between the two modalities for T_1/2_ and for the RRs at different time points. 

## 3. Results

All twelve participants tolerated the study procedures well and fully completed the study using both imaging modalities.

### 3.1. MRI

**Figure 2:**
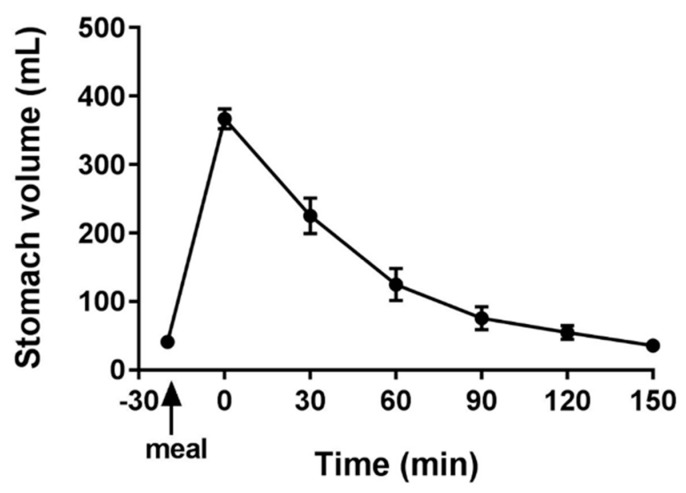
Gastric volumes measured with MRI plotted against time before and after feeding the soup test meal. *n* = 12 participants, data are shown as mean ± SEM.

The fasted baseline gastric volumes (Figure 2) showed a small amount of resting gastric juices of 41 ± 10 mL. After ingestion of the meal, volumes increased significantly (*p* < 0.0001) to reach the maximum immediately after feeding (367 ± 14 mL). This was followed by a decline in the volumes reaching the baseline at 150 min (35.5 ± 6.2 mL). The average time to empty half of the stomach contents (T_1/2_) was 44 ± 6 min.

### 3.2. GS

**Figure 3:**
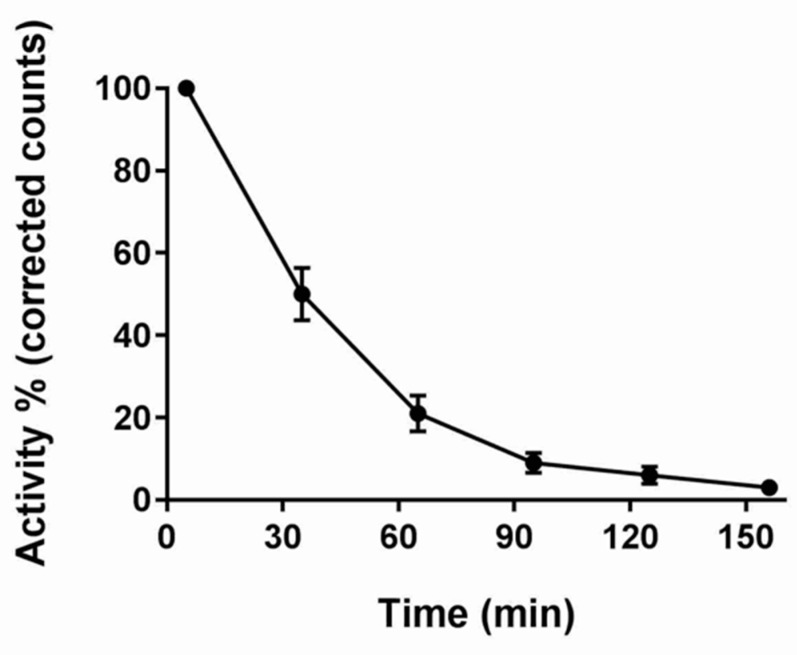
Percentage activity in the stomach region of interest measured with gamma scintigraphy plotted against time after feeding the soup test meal *n* = 12 participants, data are shown as mean ± SEM. Counts were corrected for background radioactivity and radioisotope decay.

The gastric emptying curve of *n* = 12 subjects presented by the percentage of activity in the radiolabelled meal is shown in Figure 3. The average time to empty half of the stomach contents (T_1/2_) was 35 ± 4 min, with a modest but significant difference from the T_1/2_ measured by MRI (*p* < 0.004).

### 3.3. Correlation of MRI and GS

**Figure 4:**
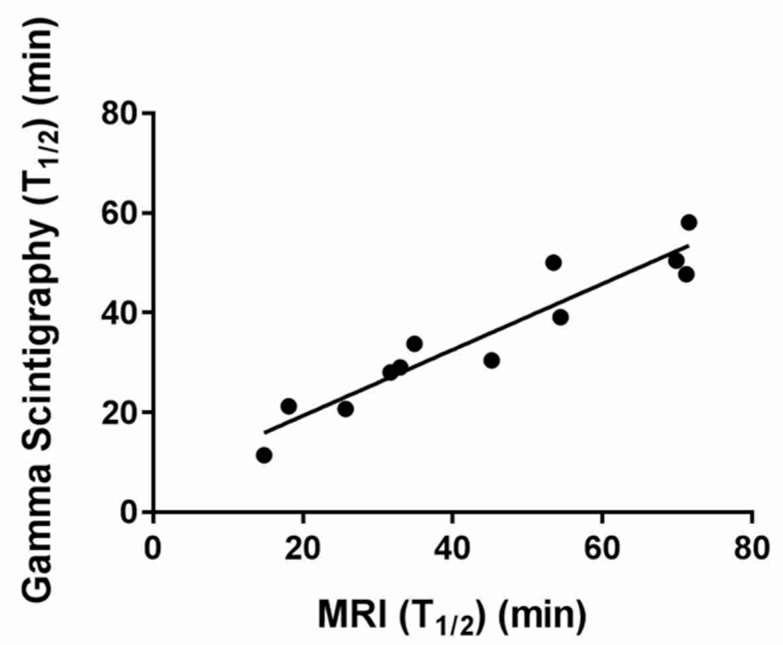
Plot of the correlation between gastric half emptying times measured by MRI and gamma scintigraphy. Each data point represents the T50% for a given participant measured by MRI and gamma scintigraphy, respectively.

We examined the strength of the correlation between the two imaging modalities using the half emptying time (T_1/2_). The correlation between MRI and GS (Figure 4) showed a good, positive correlation between both techniques (*n* = 12, Pearson’s *r* = 0.95, *p* < 0.0001). The RRs at different time points also correlated well at all time points but the last t = 150 min, with the parameters being Pearson’s *r* = 0.91, *p* < 0.00004 for RR30; Pearson’s *r* = 0.95, *p* < 0.000002 for RR60; Pearson’s *r* = 0.94, *p* < 0.00004 for RR90; Spearman’s *r* = 0.64, *p* < 0.03 for RR120; and Spearman’s *r* = 0.20, *p* = 0.54 for RR150.

## 4. Discussion

Gastric emptying is the result of harmonically coordinated accommodation and motor events, as well as neuronal and hormonal factors. Disorders of gastric emptying range from gastroparesis, dumping syndrome and dyspepsia. Gastric emptying can also be altered in patients with active inflammatory bowel disease [21]. Associated symptoms can be severe and range from discomfort to early satiety and vomiting. 

The clinical utility of gastric emptying tests has been considered in many reports and is reflected in specialist diagnostic and treatment guidelines [1,22]. A recent study has investigated the perspectives of patients with dyspeptic symptoms, who were referred for gastric emptying studies by their clinicians [23]. The study found that the clinicians perceived the gastric emptying test as useful, that it provided new diagnostic information and led to changes in management. 

A variety of techniques have been developed to assess and study gastric emptying. Suspected diagnosis of delayed gastric emptying is demonstrated using radiographic methods, gamma scintigraphy and breath tests. Each technique has limitations and disadvantages. For example, ultrasonography images suffer from the presence of air within the lumen of the gut [24] and gastrointestinal intubation studies may alter normal physiology [25].

Gamma scintigraphy is considered as the ‘gold standard’ [22]. The main advantages are the relatively simple data processing and the ability to discriminate between antral and fundal distribution and function. Some disadvantages include exposure to a small amount of ionizing radiation although, historically, there has been a lack of standardization for methods, protocols and test meals [9].

MRI has been used to measure fasting and postprandial gastric volumes [10,11,26] and most recently semi-automatic MRI analysis has been validated against gamma scintigraphy [15]. In addition, it would be expected that there may be variations in measurements of gastric emptying when measured in erect and supine positions. and there is no agreed test meal which has been adopted by either nuclear medicine or MRI communities.

To our knowledge, there are no studies whereby gastric emptying of a meal has been validated simultaneously on the same day, with both MRI and gamma scintigraphy. This study has achieved that purpose. However, we acknowledge that the study had various limitations. The test meal used is one of many test meals used for GS, but there is no agreed standard for MRI test meals. In this study, we were particularly interested in using the same soup meal that is used to elicit reliable postprandial gastrointestinal, enteroendocrine hormone peptide and appetite responses in giardia enteritis and Crohn’s disease [18,19]. Validation of the gastric emptying performance of the test meal between modalities will extend its translational use. MRI scanning was carried out in the supine position, which is different from the standing GS position. However, the soup meal remains homogeneous, thus we would not expect any possible layering effect on gastric emptying and the MRI scan time was also very brief. The modest though significantly longer time measured by MRI could reflect the ability of MRI to include the volume of orogastric secretions in the measured gastric volumes, not just the emptying of a label. The ability to image the gastric volume balance between secretion and meal emptying is an additional advantage of MRI compared to gamma scintigraphy, which can measure only the loss of ingested tracer from the stomach reservoir [15]. MRI can also show the total volume including gas, which contributes further to distension. One clear example is from aerated drinks, whereby the addition of air into the liquid as a foam can be imaged by MRI in the stomach showing that foam distends the stomach which increases satiety compared to a non-aerated drink [27].

## 5. Conclusions

In conclusion, gastric emptying was measured simultaneously by MRI and GS on the same study day for the first time, thus reducing confounding issues and variability. The time of half emptying and the retention rates correlated well. This may help with the understanding of measurements made using the different modalities and the translation of the use of this simple soup meal, known to elicit reliable physiological and pathological gastrointestinal motor, peptide and appetite responses.
